# How Do Peripheral Neurons and Glial Cells Participate in Pain Alleviation by Physical Activity?

**DOI:** 10.3390/cells14060462

**Published:** 2025-03-20

**Authors:** Menachem Hanani

**Affiliations:** 1 Laboratory of Experimental Surgery, Hadassah-Hebrew University Medical Center, Mount Scopus, Jerusalem 91240, Israel; hananim@cc.huji.ac.il; 2 Faculty of Medicine, Hebrew University of Jerusalem, Jerusalem 91120, Israel

**Keywords:** pain, physical exercise, neurons, glial cells, Schwann cells, peripheral nervous system, sensory ganglia, dorsal root ganglia

## Abstract

Chronic pain is a global health problem with major socioeconomic implications. Drug therapy for chronic pain is limited, prompting search for non-pharmacological treatments. One such approach is physical exercise, which has been found to be beneficial for numerous health issues. Research in recent years has yielded considerable evidence for the analgesic actions of exercise in humans and experimental animals, but the underlying mechanisms are far from clear. It was proposed that exercise influences the pain pathways by interacting with the immune system, mainly by reducing inflammatory responses, but the release of endogenous analgesic mediators is another possibility. Exercise acts on neurons and glial cells in both the central and peripheral nervous systems. This review focuses on the periphery, with emphasis on possible glia–neuron interactions. Key topics include interactions of Schwann cells with axons (myelinated and unmyelinated), satellite glial cells in sensory ganglia, enteric glial cells, and the sympathetic nervous system. An attempt is made to highlight several neurological diseases that are associated with pain and the roles that glial cells may play in exercise-induced pain alleviation. Among the diseases are fibromyalgia and Charcot–Marie–Tooth disease. The hypothesis that active skeletal muscles exert their effects on the nervous system by releasing myokines is discussed.

## 1. Introduction—Peripheral Versus Central Nervous System in Pain Research

In reviews on chronic pain, authors have emphasized the complexity of this topic and the variety of cellular and molecular mechanisms that underlie the diverse types of pain [1,2,3]. To this, we can add the difficulty of defining pain and the psychological and social/cultural aspects of this topic [4]. As befits such a multifaceted field, it is characterized by disagreements and controversies, which may partly arise from the different methodologies and experimental models used for research. Although the ultimate goal of most pain studies is to eventually understand chronic pain in humans and how to treat patients, experimental work has been largely performed on animals, and research on humans is limited due to ethical, financial and other reasons. The question of the applicability of animal studies to humans has been stressed by several authors [3,5,6,7,8].

One of the main controversies in the field of pain is the relative contribution of the peripheral and central nervous systems (PNS and CNS). Since the introduction of the idea of central sensitization, many investigators have strongly emphasized this approach and sometimes downplay peripheral contribution [9]. Still, a survey of the literature leads to the conclusion that several pain syndromes, some of which are severe and common, are peripherally mediated, which means that they are caused by abnormal electrical activity in sensory neurons (including the axons and somata of these neurons). Studies on animals and humans show that in many pain types, sensory neurons generate abnormally high electrical activity that activates spinal nociceptive pathways [10,11,12,13]. For example, an injection of the local anesthetic lidocaine into the dorsal root ganglia (DRG) of phantom limb pain patients greatly alleviated pain [14]. Similarly, the blockade of a peripheral nerve in patients with neuropathic foot pain resulted in a complete abolition of ipsilateral pain within 10 min [15]. See comments by Ringkamp and Raja [16] for a discussion of the strengths and shortcomings of these two studies. Voute et al. [17] summarized additional studies demonstrating that peripheral lidocaine application may be a useful therapy for treatment or pain relief in syndromes such as diabetic peripheral neuropathy and postherpetic neuralgia. In summary, studies on both humans and animals lead to the conclusion that PNS mechanisms in pain are essential in a large number of pain syndromes.

### Notes on the Terminology

1. Some authors emphasize the difference between “physical activity”, which covers all aspects of this term, and “physical exercise”, which refers to a structured and organized activity. Here, for the sake of simplicity, “exercise” refers to both types. 2. Some authors refer to the beneficial effect of exercise on pain as “exercise-induced hypoalgesia”. However, others use this term to refer to short exercise bouts that produce an immediate reduction in pain sensitivity (e.g., [18]). Another term that has been introduced is “exercise-induced analgesia (EIA)” (e.g., [19]). This term will be used here to cover all the types of physical activity.

## 2. Method

A search was performed in PubMed and covered the period up to 15 December 2024. The search terms were “exercise pain glial cells”, “exercise pain neurons”, “physical activity glial cells pain”, “hypoalgesia exercise”, “analgesia exercise glial cells”, and “myokines pain”. A total of 988 articles were found, covering studies on both humans and experimental animals. Most of the articles related to the CNS were excluded. A total of 77 articles were chosen for this review.

## 3. Physical Exercise and Pain

Chronic pain is a major global public health problem, with a prevalence of about 20% in adults [20]. Existing therapies for chronic pain are not satisfactory for many of the patients and may have serious adverse effect. This has prompted a search for non-pharmacological therapies, among which physical exercise is one of the most prominent approaches, as exemplified by the concept of “Exercise as medicine” [21,22]. The detailed mechanisms underlying the beneficial effects of exercise are largely obscure, but the role of glial cells has been emphasized. This is consistent with the understanding that in addition to the nerves, glial cells might be a target for pain therapy [23,24,25]. Again, most authors have focused on CNS glia in this context. The role of glial cells in the therapeutic effect of exercise has been reviewed recently, with an emphasis on the CNS [26]. The authors of these studies have proposed that astrocytes and microglia in the CNS are important for the generation and maintenance of chronic pain and that exercise may relieve pain by regulating these cells. The present review will discuss the possible contribution of nerve and glial cells in the PNS, with the aim of providing a more balanced view of this emerging field.

### 3.1. Confounding Factors Related to EIA

Many aspects of EIA are controversial, which may be partly due to a number of confounding factors such as the type of exercise that is studied—forced vs. voluntary exercise or aerobic vs. strength—and sex and age, which can influence the conclusions of the tests [27,28]. Also, the type, pattern, duration, and intensity of exercise can influence its health benefits [29,30,31], but these aspects have not been sufficiently investigated. Another consideration is the timing of exercise—before injury (for example, before surgery) or after it [32,33]. It was reported that prior exercise attenuated allodynia in a rat model of neuropathic pain [34]. Exercise in this model decreased neuroimmune signaling in the spinal cord and DRG and suppressed serum proinflammatory mediators. The relationship of time between the onset of injury and the start of exercise is not clear, but it has been reported that training beginning several (3-5) days after injury reduces pain [35]. Another complication is that strenuous exercise can cause pain [36] or tissue damage [37]. In this context, it is interesting to note that recent studies have shown that a small number of brief (typically 20–30 s) and intense bouts of exercise (high-intensity interval training) have profound physiological benefits, comparable to those of conventional training [38,39]. Whether such protocols, which could be easier for some patients, are superior to other types of exercise has not yet been clearly established [40,41].

The account above indicates several areas that require considerable further work: 1. There is an abundance of methods to assess pain. The standardization of these methods would help in comparing different studies and minimizing disagreements in this field. 2. Longitudinal studies on the optimal timing and protocols of exercise must be carried out. 3. Much further work needs to be performed to clarify the dependence of EIA on sex and age.

### 3.2. Neurochemical Mechanisms of EIA

The molecular mechanisms underlying EIA have been the subject of intense research for several decades. A number of hypotheses have been put forward to explain EIA, and the main one is that exercise activates endogenous neurochemical systems, which in turn act on the CNS and PNS to reduce the sensation of pain [26,27,42]. An early idea was that exercise operates via opioid agonists, which are known to act as analgesics. This was based on studies demonstrating that exercise increased the plasma levels of endogenous opioids, mainly β-endorphin [27,43]. In support of this idea, the injection of the opioid antagonist naloxone reversed at least some types of EIA in human runners [44]. Brito et al. [45] found that wheel running prevented the development of muscle hyperalgesia and that the injection of naloxone, systemically or into the brain, reversed this effect. These and other studies suggest a role for opioids in EIA, but there is no full agreement on this topic [46,47]. The opioid effects in the context of exercise are believed to occur mostly in the CNS, and the contribution of the PNS to these effects is not clear.

Catecholamines, mainly noradrenaline and adrenaline, have also been implicated in EIA and are mentioned in Section 6, which deals with the sympathetic nervous system.

The gaseous neurotransmitter nitric oxide (NO) has attracted some attention as a possible mediator of EIA [27]. However, there is considerable evidence that in some cases, NO can act as an *algetic* agent [48,49]. NO was found to activate satellite glial cells (SGCs) in DRG, thereby leading to enhanced nociception [50]. Apparently, NO acts on numerous targets, and its overall effect is not easy to predict and depends on the circumstances.

An idea that is highly relevant to the role of the PNS in EIA is that EIA is mediated by the WNT (Wingless and Int-1)-β-catenin pathway. Cho et al. [51] reported that the treadmill walking of rats improved allodynia, which was accompanied by decreased Wnt3a (a member of the WNT family) expression in DRG, compared to the sedentary group. Walking also significantly decreased β-catenin in DRG, as well as its translocation towards the nucleus of DRG neurons. Further work on the WNT-β-catenin pathway in pain is clearly warranted.

Another candidate system that may be involved in EIA are endocannabinoids (CBs), which can have analgesic effect via both the PNS and CNS pathways [52,53]. Sparling et al. [54] reported that running activated the CB system in humans and suggested a role for this system in EIA. As CB agonists affect mood and other cognitive functions, assigning a specific peripheral mechanism for these actions is not simple. Still, pursuing the role of CBs in EIA in the periphery seems an attractive direction.

Neurotrophins are an important family of growth factors and have been proposed to play a role in pain and in EIA. However, neurotrophins like brain-derived neurotrophic factor (BDNF) can have opposite actions in pain pathways [33]. On the one hand, they have been found to contribute to the generation of neuropathic conditions, and on the other hand, they promote nerve regeneration, which tends to reduce nociception. This dual effect may depend on the intensity and duration of exercise. Low- to intermediate-intensity exercise generally increases neurotrophin expression in both the PNS and CNS and is associated with neuroprotection and regeneration, whereas high-intensity exercise may lead to the lower production of neurotrophin and induce opposite effects [33].

Cytokines constitute a large group of peptides with proinflammatory and also anti-inflammatory actions, which are released mostly from immune cells and also from glial cells, adipocytes and other cells. Exercise can influence the immune system in a variety of ways: it reduces the expression of proinflammatory cytokines, increases anti-inflammatory cytokine levels and positively modulates the state of the immune system. By these actions, it can relieve pain [55,56,57], but clear evidence for the role of cytokines in EIA in humans is largely lacking. For example, in a review on rheumatoid arthritis (RA), it was concluded that following exercise, inflammatory markers and inflammatory cytokines were not different between people with or without RA [58].

In summary, the brief account above is far from exhaustive, and the reader is referred to articles dedicated to each of these (and other) systems. It should be noted again that it would not be easy to assign a specific role in EIA for substances such as NO and noradrenaline as these agents have a wide range of actions on the nervous system, some of which can be apparently antagonistic. Another family of chemical messengers are myokines, which are discussed below in some detail, because, mechanistically, they appear to be more relevant to the PNS than most of the systems mentioned above.

### 3.3. Myokines, Exercise, and Pain

During exercise, the body undergoes a cascade of physiological changes to meet the increased demand for energy and performance. One fascinating aspect of this process is the release from various organs of factors designated as “exerkines”. An important subtype of exerkines are myokines—short peptides belonging to the cytokine family, which are released from active skeletal muscles. We will focus here on myokines as they play crucial roles in mediating the communication between different organs and tissues, contributing to the systemic benefits of exercise beyond just muscle adaptation.

Myokines, e.g., interleukin-6 (IL-6), IL-15, and myostatin can have anti-inflammatory effects on various organs, like fat tissue and the liver [59]. Irisin is a well-studied myokine with numerous actions, including being an anti-inflammatory agent [60]. Rahman et al. [61] reported that irisin reduced mechanical allodynia and thermal hyperalgesia in complete Freund’s adjuvant (CFA)-treated mice. In correlation with this, it decreased the level of the proinflammatory cytokines IL-1β and tumor necrosis factor-α (TNF-α) and increased the expression of anti-inflammatory ones (IL-4 and IL-10). In the DRG, irisin also reduced the activation of spinal cord astrocytes. The effect on SGCs was not reported, but it is possible that irisin has a similar effect on these cells as well, as activated SGCs in sensory ganglia release proinflammatory cytokines [62]. It can be proposed that the release of irisin from active muscles during exercise may alleviate pain by the action of glial cells in both the spinal cord and DRG.

Another myokine with potential for pain therapy is meteorin, which was found to have a robust and sustained antinociceptive effect in a rodent model of chemotherapy-induced pain [63]. These authors also found that meteorin reduced the expression of the gap junction protein connexin 43 (Cx43) in SGCs in mouse DRG. Augmented gap junctions in SGCs is one of the hallmarks of SGC activation [24,64], and the authors therefore stated that meteorin’s analgesic effects involve SGC deactivation. However, they did not examine changes in the activation marker glial fibrillary acidic protein (GFAP), and therefore, this point needs to be verified. Still, this work clearly indicated that the myokine meteorin has an analgesic effect, which may be mediated by SGCs, supporting the notion of myokines as a link between exercise and analgesia.

In summary, myokines may be a major link between muscle activity and EIA, but clearly, this idea requires further research in both humans and in animal models. Besides the basic interest in this topic, it may have therapeutic significance. One challenge with exercise is that many people avoid it out of choice—the so-called “kinesiophobia” (fear of movement), [65]—or are unable to do it, and this holds even more strongly for those who suffer from chronic pain [55]. Thus, identifying chemical agents for treating chronic pain is of considerable importance, and myokines or drugs that mimic their actions could be practical pain medicines. It may be proposed that SGCs could be highly suitable targets for such treatments because, unlike the CNS, in sensory ganglia, blood vessels are permeable to peptides and proteins [66].

**Figure 1:**
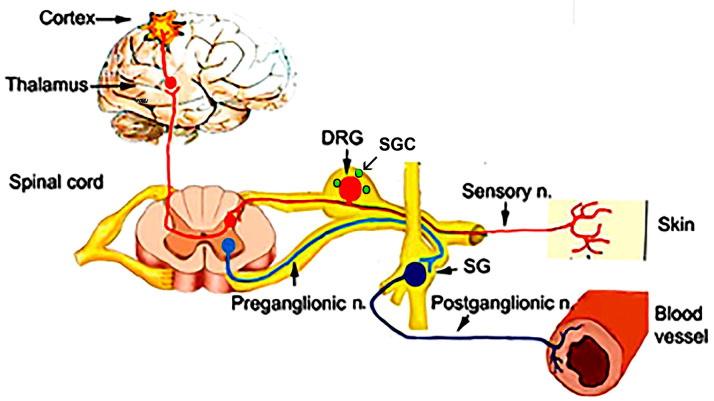
The organization of the somatosensory pathways. Neurons in dorsal root ganglia (DRG) send one axon, one branch of which innervates various body parts, including the skin, and another branch enters the spinal cord. These neurons are surrounded by specialized glial cells—satellite glial cells (SGCs), labeled green. SG, sympathetic ganglion.

In the sections below, various structures in the PNS will be discussed in the context of EIA. Figure 1 is a schematic showing the main somatosensory pain pathways, in which the DRG is a key element. The main cell types in sensory ganglia are the neurons, myelinated and non-myelinated axons, and satellite glial cells (SGCs).

## 4. Sensory Ganglia and Exercise

In view of the role of sensory ganglia in pain states (see above), it is worthwhile asking whether exercise can exert its effects on these ganglia, thereby contributing to EIA. A study on rats showed that early treadmill training after nerve injury reduced pain behavior and also decreased the level of nerve growth factor (NGF) and BDNF expression in sensory ganglia [27,33]. This was ascribed to the inhibition of the sprouting of nerves in the skin. In contrast, in another study, it was found that exercise enhanced the regrowth of DRG axons after nerve crush injury and that this effect was apparently due to a high level of neurotrophin 3 in the ganglia [67]. Cho and Seo [68] induced sciatic nerve injury in rats and found that walking exercise relieved mechanical allodynia. Walking also increased the levels of growth associated with protein 43 and BDNF in DRG; these factors promote nerve regeneration and reduce levels of proinflammatory cytokines. Thus, exercise has several actions on DRG that can counter the effect of nerve injury, indicating that sensory ganglia are an important component of EIA. Almeida et al. [69] found that exercise alters levels of neurotrophins in DRG, inducing an effect that can transpire as either their up- or down-regulation, which fits the idea of the dual role of neurotrophins in nerve injury [33], but this issue must be further clarified.

### 4.1. Satellite Glial Cells (SGCs)

The main type of glial cells in sensory ganglia are SGCs [24,66,70,71,72,73,74]. SGCs form a complete sheath around the sensory neurons, with a narrow (20 nm) gap between neurons and glia, allowing effective SGC-neuron interactions. Peripheral nerve injuries such as axotomy or inflammation upregulate the level of the activation marker GFAP in SGCs [73] and increase cytokine content in them [62]. These changes are consistent with SGC activation (gliosis). Additional changes that take place upon SGC activation include the following: 1. Potassium Kir4.1 channels in SGCs are suppressed, which may contribute to pain [75]. 2. The sensitivity of SGCs to the pain mediator ATP increases [76,77]. 3. Gap junction-mediated coupling among SGCs increases after injury, which appears to be part of the gliosis process [78]. The injection of gap junction blockers, or the neutralization of the gap junction protein in SGCs, reduced pain behavior in several rodent pain models [79,80,81].

These findings have led to the conclusion that SGCs should be considered as important contributors to chronic pain and can be targeted for pain treatment [24,70]. Furthermore, sensory ganglia, unlike most CNS regions, lack a vascular barrier and are therefore exposed to the contents of the circulation, and thus, the position of SGCs around the neurons makes them an ideal target for pain therapy. There is emerging information that SGCs may underlie various pain syndromes in humans, such fibromyalgia [82]. For a review on this topic, see [70].

**Figure 2:**
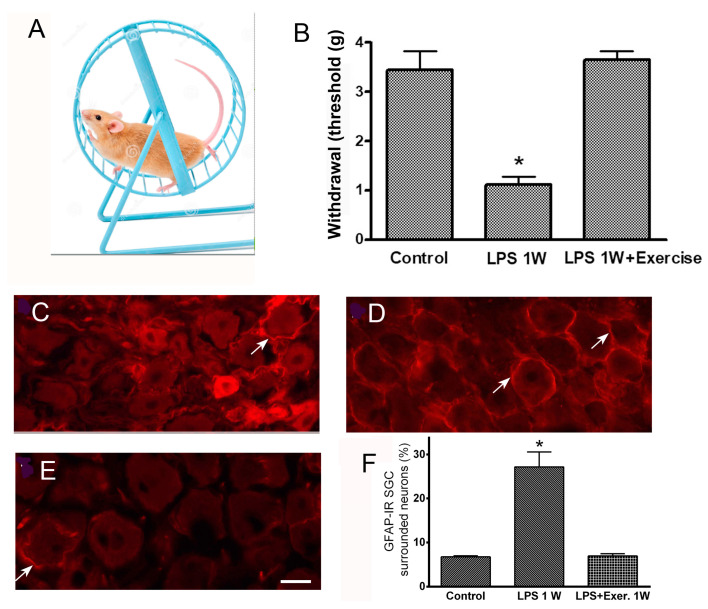
Exercise reduces SGC activation and has an analgesic effect in mice with systemic inflammation induced by lipopolysaccharide (LPS). (**A**), the wheel used for voluntary exercise. The mice ran 2–3 km/day. (**B**), running raised the threshold of withdrawal in response to the mechanical stimulation of the hind paw with von Frey filaments. The mice received a single injection of LPS (2.5 mg/kg) and were placed in a cage with the wheel. One week later, the threshold was back at the control level and was much higher than the value for the LPS-treated mice that did not run. C-E, the immunostaining of DRG sections for the glial activation marker glial fibrillary acidic protein (GFAP). (**C**), control. Very few neurons are surrounded by immunopositive SGCs; a neuron surrounded by GFAP+ SGCs is indicated with an arrow. (**D**), one week after LPS injection, many more neurons are surrounded by GFAP+ SGCs. Several GFAP+ SGGs are indicated with arrows. (**E**), the DRG section from a mouse that received LPS and ran for 1 week. Note the small number of GFAP+ SGCs. Calibration bar, 20 µm. (**F**). A quantitative summary of the immunostaining results. Modified from [83]. Asterisks in B and F indicate *p* < 0.05.

A recent study addressed the possible contribution of SGCs to EIA in a mouse model of chronic pain based on systemic inflammation induced by a single injection of lipopolysaccharide (LPS) [83]. An earlier investigation showed that LPS induced mechanical hypersensitivity in mice and that, in parallel, SGCs in DRG were activated, as assayed by augmented GFAP immunostaining [77]. LPS also caused increased SGC-SGC and neuron–neuron coupling by gap junctions, which was demonstrated by the dye coupling method and electron microscopy. In the work mentioned above [83], voluntary wheel running was started on the day of LPS injection and lasted for one week; the results were compared to those from mice that received LPS but did not exercise. This comparison showed that a week of exercise reversed all the parameters that were measured: tactile sensitivity returned to normal, GFAP immunostaining was back at the control level, and SGC and neuronal coupling also returned to their control values (see Figure 2). It was concluded that exercise reduced pathological changes in DRG that were previously found to be associated with augmented pain behavior. As SGC activation was found to contribute to pain [24,81], the finding that exercise reduced both pain behavior and reversed SGC activation suggests that one possible mechanism for EIA involves changes in SGCs. Further work needs to be conducted along these lines, using other pain models and additional modes of exercise. Finding ways to reverse SGC activation appears to have potential for pain therapy.

Diabetes mellitus (DM) has become a global epidemic, affecting hundreds of millions of people of all ages. A major complication in DM is diabetic peripheral neuropathy (DPN), afflicting at least 50% of patients [84], and it is frequently associated with pain. There is evidence that functional changes in sensory neurons contribute to diabetic pain. In animal models of DM, sensory neurons show a misexpression of Na^+^ channels [85] as well as numerous molecular abnormalities [86], indicating that sensory ganglia are highly suitable targets for research into and the therapy of DPN [87]. Exercise has been recommended as one of the main non-pharmacological therapies for DPN [88]; however, it is not easy to implement this in practice. In view of the role of SGCs in pain, it is conceivable that exercise deactivates them in DPN, as found in other pain states. Finding out what the molecular targets of exercise in these cells are could lead to the development of agents that can mimic the actions of exercise and serve as pain therapy in DPN.

As emphasized above, the activation of SGCs in the sensory ganglia plays a crucial role in neuropathic pain through the release of proinflammatory mediators and by other mechanisms. Hanani et al. [89] induced a model of DM type 2 in mice and rats by streptozotocin (STZ) injection. SGCs in the DRG of these animals were activated, as evidenced by GFAP upregulation, which was accompanied by tactile hypersensitivity. Wang et al. [90] used the STZ model in rats and observed mechanical and thermal hyperalgesia. SGCs in these rats showed a higher expression of P2Y12 purinergic receptor (P2Y12R) messenger RNA (mRNA) and protein compared to the controls. IL-1β expression levels in the DRG were also increased in the DM rats. Targeting P2Y12R by short hairpin RNA (shRNA) decreased the hyperalgesia, the upregulated expression of P2Y12R mRNA and protein, GFAP, and IL-1β. These results indicate that the upregulation of P2Y12R is part of the SGC activation in this model and that these receptors may be a target for pain relief in DM. It would be interesting to find out whether P2Y12Rs are influenced by exercise.

#### SGCs and Immune Disease

Autoimmune diseases, such as multiple sclerosis (MS) and rheumatoid arthritis (RA), are characterized by pain that is difficult to treat [55,91]. Exercise is highly recommended in patients with immune diseases, which agrees with the growing evidence that physical activity curbs inflammation [55].

Recent work has revealed the unexpected involvement of SGCs in autoimmune diseases and their possible contribution to pain in these diseases. The injection of autoantibodies from RA patients into mice induced pain behavior in these animals [92]. Moreover, the RA autoantibodies were found to bind selectively to SGCs, and with additional stimuli like LPS, enhanced transcriptional changes in DRG cells and released pronociceptive factors from SGCs. Although fibromyalgia is not defined as autoimmune disease, it was found the autoantibodies from fibromyalgia patients had similar effects on SGCs (of mice and humans) to those found for RA and also activated SGCs [82]. These findings suggest that SGCs are key elements in the development pain in these diseases. In light of the evidence that exercise is highly beneficial in autoimmune diseases [55,93], it would be highly interesting to find out whether SGCs are involved in the beneficial actions of exercise in these diseases. Some evidence in this direction was obtained by Warwick et al. [94], who used the experimental autoimmune encephalomyelitis (EAE) model of MS in mice. They found that SGCs in the DRG of EAE mice were activated, in parallel with augmented pain behavior. In view of the reversal of SGC activation by wheel running in LPS-treated mice [83], it might be expected that exercise would affect SGCs in a similar manner in MS and other immune diseases, thus contributing to pain alleviation. Clearly, this idea needs to be tested experimentally, but this area appears to be a highly promising avenue for research in both humans and animals.

Information on the diseases discussed here is presented in Table 1.

## 5. Axons and Schwann Cells (SCs)

All peripheral axons are associated with SCs, which either wrap axons by forming a myelin sheath or surround axons as non-myelinating SCs. This intimate association between nerves and SCs is consistent with the essential roles that SCs play in the normal functions of nerves and also suggests that pathological changes in SCs result in neuronal dysfunctions, among which pain may be a prominent feature [3,104,105,106]. Unlike the nerves in the CNS, peripheral nerves can regenerate after injury [107,108]. This appears to be mainly due to the presence of SCs, which promote myelination and release growth factors. Goulart et al. [109] showed that the combination of treadmill training and grafting of SCs improved sciatic nerve regeneration following sciatic nerve injury in mice. Thus, it can be proposed that an important effect of exercise is to act in concert with SCs to enable nerve regeneration. As nerve injury is a major factor in pain generation, nerve repair should contribute to EIA. Two neurological disorders that are associated with SC dysfunction are briefly discussed below.

### 5.1. Charcot–Marie–Tooth Disease

Charcot–Marie–Tooth disease (CMT) is a group of hereditary diseases that affect both sensory and motor nerves in the limbs. The affected nerves degenerate slowly and lose the ability to communicate with their targets, resulting in muscle weakness and atrophy in the arms, legs, hands, or feet. Pain is reported by 23–85% of CMT patients [95]. Exercise has been suggested as beneficial for CMT patients, but the mechanism underlying this effect has not yet been identified. Some insight into this question has been obtained in studies on pain models in animals. Among CMT types, CMT1 is the most common and is caused by abnormalities in the myelin sheath. Klein et al. [110] used mutant mice that displayed CMT1 symptoms and tested the effect of wheel running in two groups: one group ran freely, and in the other, running was limited by 40%; these mice were compared to CMT1 mice that did not run. Klein et al. [110] found an increase in the number of macrophages in the quadriceps nerves of CMT1 mice. They also identified abnormal myelin and axonal damage in these mice. Electrophysiological recordings revealed an expected reduction in nerve conduction velocity (NCV) in CMT1 mice and a reduction in motor performance. Both limited and unlimited exercise reversed the elevation in macrophage numbers and led to less axonal damage and better motor performance. Also, both exercise types significantly improved NCV in comparison to the control mutants (non-running). Interestingly, only limited exercise reduced the myelin-related pathological features and preserved myelin thickness compared to the control mutants. This study indicates several mechanisms by which exercise provides beneficial effects in CMT. Although Klein et al. [110] did not address the pain issue directly, it is possible that the improvements in motor functions are related to pain alleviation. Also, it is clear that an improvement in SC structure and function is an essential part of exercise in this model. It would be of interest to find out whether the improvement in myelin is related to EIA, which was observed in CMT patients. This study confirms other reports that intense exercise may not have universally beneficial health effects.

### 5.2. Guillain–Barré Syndrome

Guillain–Barré syndrome (GBS) is an autoimmune disorder where the axons of spinal motor neurons are injured. In one variant of GBS, there is the demyelination of these axons, which results in muscle weakness [96,97]. Pain is common in GBS, and exercise has been found to improve its symptoms, including pain [111], but information on this topic is scarce.

## 6. The Enteric Nervous System

The intrinsic ganglia of the digestive system—the enteric nervous system (ENS)—is a complex and largely autonomous network of neurons and nerve fibers that regulates most of the digestive functions [112]. The ENS consists of two divisions—the myenteric and submucosal plexuses. The main types of glia in the ENS are enteric glial cells (EGCs), which are specialized cells that are similar in several respects to astrocytes [113,114,115]. Under various injuries (e.g., bacterial and viral infections), EGCs can undergo reactive gliosis, which is a largely deleterious response, where EGCs display proinflammatory activity and can further contribute to gastrointestinal (GI) disturbances. For example, it was shown that EGCs mediate HIV-1-induced diarrhea by sustaining intestinal neuroinflammation through mechanisms involving toll-like-receptor-4 (TLR4), glial S100β, inducible NO synthase (iNOS), and NFkB activation [98,113].

There is emerging information on the possible contribution of EGCs to pain. Grubišić et al. (2020) [116] reported that EGCs in both mice and humans regulate visceral hypersensitivity during chronic intestinal inflammation by mechanisms involving the gap junction protein connexin 43 (Cx43) in EGCs and macrophage colony-stimulating factor 1 (CSF1). Deleting Cx43 in EGCs protected against visceral hypersensitivity in a colitis model but did not influence the progression and severity of the disease. This suggests that preventing EGC gliosis might be a target for treating visceral pain. Further work [99] has supported these findings, suggesting the role of prostaglandin E2 during EGC activation.

Parkinson’s disease (PD) manifests in motor deficiencies and is associated with changes in the basal ganglia in the brain, but there appears to be a link between this disease and the ENS. In addition to the well-recognized motor dysfunctions, PD patients suffer from GI problems, mainly constipation and abdominal pain [100,101,117]. There is evidence that exercise is beneficial to GI function, but the underlying mechanisms are largely unknown [118,119]. O’donovan et al. [120] generated a PD model in mice and found, among other changes, decreased submucosal neuronal density and the augmented expression of the glial marker S100β in the myenteric plexus (indicating intestinal inflammation). These animals were divided into two groups: one was sedentary, and the other performed voluntary running. The authors found that running protected against submucosal neuronal changes but had no effect on S100β expression. On the one hand, these results confirm that there is a link between PD and the ENS and demonstrate the beneficial effect of exercise on ENS neurons. On the other hand, based on the negative S100β results, exercise did not improve the inflammatory changes in EGCs. Still, this work showed that a model of PD displays gut pathology, and clearly, this line of study should be pursued further.

## 7. The Peripheral Sympathetic Nervous System

The sympathetic nervous system (SNS) has been implicated for many years in chronic pain, and blocking sympathetic transmission has been considered as a treatment modality for patients whose pain was refractory to conservative therapy [121]. The main function of the peripheral SNS is innervating effectors such as smooth muscles, cardiac muscle and glands, and it is therefore considered as a largely motor system. However, the release of neurotransmitters from sympathetic terminals may have additional roles. The SNS can be blocked by ablating sympathetic nerves (sympathectomy), which is performed chemically, surgically, or by other methods [122]. Alternatively, local anesthetics have been used to block sympathetic nerve conduction. However, evidence in support of the efficacy of sympathectomy as pain therapy has not been strong [121].

A pain syndrome that is believed to be associated with the SNS is Complex Regional Pain Syndrome (CRPS), which is a debilitating disorder caused by trauma—usually bone fracture in the limbs. Sympathetic block has been suggested as a potential treatment for CRPS, but evidence of its efficacy is not sufficiently strong [123]. There is some information on the effect of exercise in animal models of CRPS, where a bone (usually the tibia) is broken, and pain behavior and other parameters are measured after the cast is removed. It was found that exercise had a beneficial effect on pain behavior in these animals [124,125]. Exercise also reduced edema in the leg, which is a key clinical characteristic in CRPS patients [125]. The mechanism underlying these effects was not fully clarified, but it was proposed that exercise reduced the level of neurotrophins, which contribute to the pain in CRPS [125]. These animal studies suggest that exercise may be considered as an effective therapy for CRPS patients; however controlled studies on humans still need to be performed. Moreover, CRPS patients usually prefer to avoid physical activity, which hinders such studies. How glial cells are involved in these effects still needs to be explored, but pursuing this topic is highly promising.

Another type of CRPS model is the ischemia and reperfusion of a limb. De Logu et al. [102] used this model in mice and found that pain was associated with pathologic changes in the tibial nerve, accumulation of macrophages, and transient receptor potential type ankyrin1 channel (TRPA1) in Schwann cells. Damage to cutaneous sympathetic nerves was also identified in CRPS patients [103].

Another possible piece of evidence for the role of the SNS in chronic pain is the finding that nerve injury induces the sprouting of sympathetic nerve fibers in DRG [126]. However, clear evidence for the contribution of this sprouting to pain is not available. A recent study on mice showed that after nerve injury, DRG neurons are occasionally fired spontaneously in clusters in the spared nerve injury (SNI) of the sciatic nerve [127]. This firing was triggered by the activity of sympathetic nerves, which indeed were found to sprout in DRGs in this pain model. The authors identified noradrenaline as a key neurotransmitter mediating this firing and concluded that inhibiting sympathetically mediated cluster firing may be used for treating spontaneous pain. This and other studies provide support for the concept of sympathetically related pain, but the role of glial cells in this phenomenon remains to be investigated.

Physical activity is associated with augmented sympathetic activity, and it may be expected that this could lead, at least partly, to a link between exercise and hypoalgesia. However, there is little information on this topic, which is surprising because it is well established that exercise activates the SNS, leading to the release of noradrenaline and adrenaline from sympathetic nerves and the adrenal medulla, respectively [128]. This account presents a paradox. On the one hand, there is some evidence that blocking the SNS can relieve pain. On the other hand, SNS activity increases during exercise, which would counter the established analgesic actions of exercise. Thus, the SNS may have a dual effect on pain, which could explain the uncertainties that were alluded to above on the effect of SNS activity in pain. To make progress in this field, these two opposing actions need to be investigated, and the role of neurons, glia and endocrine cells must also be better understood at both the central and peripheral levels.

## 8. Conclusions

The main aim of this review was to discuss some ideas on how exercise can relieve pain via actions on the PNS. The reader has very likely noticed the paucity of concrete information on this topic, but looking at the positive side, there is firm support for the proposition that exercise has numerous health benefits, including pain relief. The mechanisms underlying EIA are still largely obscure, at least partly because of numerous confounding factors involved in pain and exercise research. Still, in view of the manifold roles of glial cells in the PNS, understanding the normal physiology and pathophysiology of these cells better can clarify how EIA works. The author hopes that this review has pointed out several promising research directions that will help in achieving this goal and will lead to the development of better exercise-related pain therapies.

## Figures and Tables

**Table 1:** A summary of the disorders characterized by pain, and the types of peripheral nervous system cells that might be involved in the pain symptoms.

Name of Disorder	Suggested Peripheral Cells Involved	Comment	References
Fibromyalgia	Satellite glia, nerve fibers	Human immunoglobulins tested in mice	[82]
Multiple sclerosis	Satellite glia	Mouse model	[94]
Diabetes	Satellite glia	Rat model	[90]
Rheumatoid arthritis	Satellite glia	Mouse model	[92]
Charcot–Marie–Tooth	Schwann cells	Humans	[95]
Guillain–Barre	Axons	Humans	[96,97]
Intestinal inflammation	Enteric glia	Mouse models	[98,99]
Parkinson’s disease	Enteric neurons and glia	Humans, rodent models	[100,101]
Complex Regional Pain	* Schwann cells, * macrophages # Sympathetic nerves	* Mouse model, # humans	[102] *, [103] #
Systemic inflammation	Satellite glia	Mouse model	[77]

* indicates that reference [102] is a study on mice, and # indicates that reference [103] is on humans.

## Data Availability

No new data were created or analyzed in this study.

## Abbreviations

The following abbreviations are used in this manuscript:

BDNF	Brain-derived neurotrophic factor
CMT	Charcot–Marie–Tooth disease
CNS	Central nervous system
CRPS	Complex Regional Pain Syndrome
Cx43	Connexin 43
DM	Diabetic mellitus
DPN	Diabetic peripheral neuropathy
DRG	Dorsal root ganglion
ECB	Endocannabinoids
EAE	Experimental autoimmune encephalomyelitis
EGC	Enteric glial cell
EIA	Exercise-induced analgesia
ENS	Enteric nervous system
GBS	Guillain–Barré syndrome
GFAP	Glial fibrillary acidic protein
GI	Gastrointestinal
IL-6	Interleukin-6
iNOS	Inducible NO synthase
LPS	Lipopolisaccharide
NCV	Nerve conduction velocity
MS	Multiple sclerosis
NO	Nitric oxide
PD	Parkinson’s disease
PNS	Peripheral nervous system
RA	Rheumatoid arthritis
SC	Schwann cells
SGC	Satellite glial cell
SNI	Spared nerve injury
SNS	Sympathetic nervous system
STZ	Streptozotocin
TLR4	Toll-like-receptor-4
TNF-α	Tumor necrosis factor-α
TRPA1	Transient receptor potential ankyrin1 channel
WNT	Wingless and Int-1
